# Azole fungicides and *Aspergillus* resistance, five EU agency report highlights the problem for the first time using a One Health approach

**DOI:** 10.1038/s44259-025-00140-0

**Published:** 2025-10-23

**Authors:** Michaela Lackner, Ana Alastruey-Izquierdo, Darius P. H. Armstrong-James, Michael J. Bromley, Matthew C. Fisher, Paul E. Verweij

**Affiliations:** 1 Medical University of Innsbruck, Institute of Hygiene and Medical Microbiology, Innsbruck, Austria; 2 MYCOS - PhD Training Program, Medical University of Innsbruck, Innsbruck, Austria; 3 Mycology Reference Laboratory, National Centre for Microbiology, Instituto de Salud Carlos III, Madrid, Spain; 4 Center for Biomedical Research in Network in Infectious Diseases (CIBERINFEC), Instituto de Salud Carlos III, Madrid, Spain; 5 Department of Infectious Disease Epidemiology, Imperial College London, London, UK; 6 Manchester Fungal Infection Group, Division of Evolution, Infection, and Genomics, Faculty of Biology, Medicine and Health, University of Manchester, Manchester, UK; 7 Medical Research Council Centre for Global Disease Analysis, Imperial College London, London, UK; 8 Department of Medical Microbiology and Radboudumc Center of Expertise for Mycology, Radboud University Medical Center, Nijmegen, The Netherlands; 9 Center for Infectious Disease Research, Diagnostics and Laboratory Surveillance, National Institute for Public Health and the Environment (RIVM), Bilthoven, The Netherlands

**Keywords:** Drug safety, Agriculture, Fungal infection, Environmental sciences

The European Food Safety Agency (EFSA) recently published a report on the impact of azole fungicides on azole resistance in *Aspergillus* species, issued by the European Commission, and involving for the first time a multiagency collaboration including the European Centre for Disease Prevention and Control, the European Chemicals Agency, the European Environment Agency, and the European Medicines Agency, with support of the European Commission’s Joint Research Centre^1^.

Resistance selection through azole fungicide exposure has been well documented in *Aspergillus fumigatus*, which is the primary cause of *Aspergillus* diseases in humans^2^. Certain triazole fungicides show activity against *A. fumigatus*, which is not a plant pathogen, but a saprophyte that is found in plant-and timber-waste piles^3^. Residues of azole plant protection products in the green-waste select for resistance, most dominantly due to two signature mutations in the Cyp51A target gene, known as TR_34_/L98H and TR_46_/Y121F/T289A^4^. These substitutions render medical azoles ineffective, contributing to excess mortality in patients who contract azole-resistant invasive aspergillosis^5^.

The EFSA report is comprehensive and provides details on azole fungicides that have been authorized for use in European member states, examines evidence that supports a causative link between azole fungicide use and the development of azole-resistant *Aspergillus* disease, reviews drivers for environmental resistance selection, and proposes a risk assessment framework for current and new authorizations of azole fungicides.

Approximately 10,000 tonnes of azole fungicides for plant protection are sold annually in the EU, accounting for >99% of azoles sold, with azole biocides, industrial azoles, and medical/veterinary azoles making up the remainder. The Netherlands and Germany rank as the top countries with respect to fungicide sales per hectare of arable land, but conclusions at a national level are inaccurate as use data were not available, and some member states refrained from providing data. Despite stable annual fungicide sales, accumulation of azoles in the environment is likely due to their long half-life in soils, posing unknown risks to human, animal, and ecosystem health^6^. To study environmental selection pressures, 12 fungicides were further assessed based on approval status and activity against *Aspergillus*. Risk assessment of environmental resistance selection was based on agricultural processes that produce waste streams that support the growth of *A. fumigatus*, and the presence of an azole selection factor, estimated by assessing the fungicide residue levels in relation to the predicted no effect concentrations for resistance (PNEC_res_) derived from laboratory experiments. This analysis resulted in a heat map of potential hotspots of resistance selection for various agricultural production processes. Furthermore, a preliminary risk assessment framework for compound authorization was presented using a tiered approach^1^.

The report lists no less than 23 main priority data gaps, including lack of use-data of fungicides, which is a key factor in antifungal stewardship. It also highlights 29 recommendations to address these data gapsMethodology to assess risk of resistance selection based on PNEC_res_ requires further validation and refinement including calculation of PNEC_res_ in conditions that better mimic natural environments and that consider multiple fungicide exposure^7^. Better understanding of resistance transmission and spread is required as well as One Health surveillance programs, application of genomics to unravel transmission dynamics, and determination of azole residue levels in food, surface water, and the environment. As the activity of environmental fungicides against human pathogens is not currently part of the authorization process, regulatory actions are needed along with validation of the preliminary risk assessment framework (Fig. 1).

**Fig. 1:**
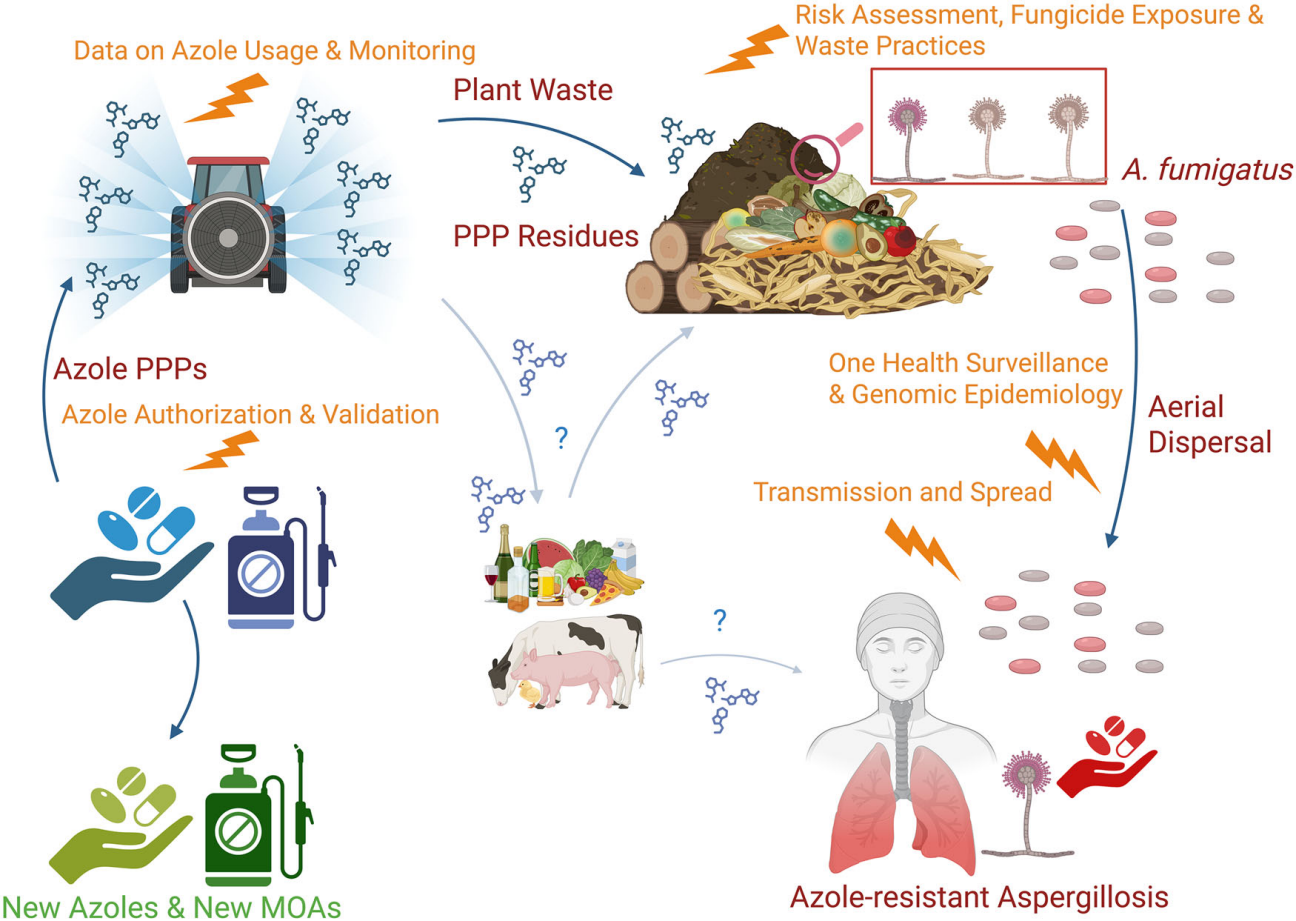
Recommended actions (orange lightning bolts) to address key knowledge and data gaps on azole usage, contamination, and resistance hot spots, as outlined in the EFSA report^1^. The recommended actions span the entire process of fungicide use, including compound authorization, application, agricultural waste management, and transmission pathways to humans. Blue arrows illustrate interactions between different fields and the spread of azole fungicides. Blue question marks highlight areas of uncertainty regarding the spread of plant protection products (PPPs) and their potential indirect health impacts through agricultural products and the food chain. Blue pills represent azoles used as PPPs, green pills indicate novel medicines and PPPs, and red pills denote currently used medical azoles. Beige *Aspergillus* species and spores are azole-susceptible, while red *Aspergillus* species and spores are azole-resistant. The figure also depicts the aerial dispersal of *Aspergillus* spores and the subsequent infection of a patient with azole-resistant aspergillosis. PPP plant protection product, *A. fumigatus* *Aspergillus fumigatus*, MOA mechanism of action.

While in 2002 the EU Scientific Steering Committee concluded that the increased azole treatment failure of fungal infections with azole antimycotics was not related to the use of azole fungicides in agriculture, the current EFSA report is a call for action^8^. Action is urgently needed given the recent authorizations of ipflufenoquin for plant protection, which shares its novel mode of action (dihydroorotate dehydrogenase inhibitor) with olorofim, an antifungal currently in phase-III clinical trial evaluation for (azole-resistant molds including *A. fumigatus*) fungal diseases. Successful selection of olorofim resistance in *A. fumigatus* through exposure to ipflufenoquin underscores the dual-use cross-resistance selection potential^9^.

The EFSA report was requested by the European Commission, and we understand it as a clear call for action towards researchers and funding organizations to address the knowledge gaps and help contain azole pollution and drug resistance selection in fungi. An interdisciplinary approach is needed to find sustainable solutions that allow us to ensure food security for an ever-growing human population, alongside preserving effective antifungal treatments for our at-risk patient cohort (e.g., elderly, neonates, immunocompromised and immunosuppressed hosts). There has been an increase in networked activity both within and outside Europe that includes academic, industry, and policymakers with the potential to build roadmaps aimed at mitigating the threat of emerging fungal antimicrobial resistance^10^.

## Data Availability

No datasets were generated or analyzed during the current study.
